# Building a Bridge to Community: A Pragmatic Randomized Trial Examining a Combined Physical Therapy and Resistance Exercise Intervention for People after Head and Neck Cancer

**DOI:** 10.3390/cancers16091758

**Published:** 2024-05-01

**Authors:** Margaret L. McNeely, K. Ming Chan, Ryan A. Spychka, Joni Nedeljak, Brock Debenham, Naresh Jha, Hadi Seikaly

**Affiliations:** 1 Department of Physical Therapy, Faculty of Rehabilitation Medicine, University of Alberta, Edmonton, AB T6G 2G4, Canada; spychka@ualberta.ca (R.A.S.); jnedelja@ualberta.ca (J.N.); 2 Department of Oncology, Faculty of Medicine and Dentistry, University of Alberta, Edmonton, AB T6G 1Z2, Canada; brock.debenham@albertahealthservices.ca (B.D.); naresh.jha@albertahealthservices.ca (N.J.); 3 Department of Physical Rehabilitation Medicine, University of Alberta, Edmonton, AB T6G 2E1, Canada; kming@ualberta.ca; 4 Department of Radiology & Diagnostic Imaging, University of Alberta, Edmonton, AB T6G 2B7, Canada; 5 Department of Radiation Oncology, Cross Cancer Institute, Alberta Health Services, Edmonton, AB T6G 1Z2, Canada; 6 Division of Otolaryngology-Head & Neck Surgery, University of Alberta, Edmonton, AB T6G 2B7, Canada; hadi.seikaly@albertahealthservices.ca

**Keywords:** physical therapy, exercise, quality of life, fatigue, randomized controlled trial, survivorship

## Abstract

**Simple Summary:**

Head and neck cancer is associated with significant morbidity. Shoulder and neck dysfunction, weight loss, and declines in muscular strength and physical functioning are commonly seen following cancer therapies. This pragmatic randomized controlled trial involved sixty-one individuals with cancer in the head and neck region who had completed their cancer treatment. The primary aim was to assess the effectiveness of the addition of lower-body resistance exercise training to a physiotherapeutic shoulder and neck exercise protocol on fatigue-related quality of life. Findings support the benefit of the combined intervention for fatigue-related quality of life, six-minute walk test distance, upper- and lower-body muscular strength, and physical activity level. The continued improvement in treatment effect over the one-year follow-up period suggests promise from this interdisciplinary approach to bridge individuals with head and neck cancer from rehabilitation care to general exercise and physical activity.

**Abstract:**

Background: Established barriers to general exercise and physical activity among individuals with head and neck cancer include dry mouth, difficulty eating, weight loss, fear of injury, comorbidities, and treatment-related symptoms of pain and fatigue. Methods/Design: A 12-week pragmatic randomized controlled trial was conducted followed by an optional supported exercise transition phase. Eligible participants were individuals with head and neck cancers who had undergone surgery and/or radiation therapy to lymph node regions in the neck. Participants were randomized to a comparison group involving a shoulder and neck physiotherapeutic exercise protocol, or to a combined experimental group comprising the shoulder and neck physiotherapeutic exercise protocol and lower-body resistance exercise training. The primary outcome of this study was fatigue-related quality of life. Results: Sixty-one participants enrolled, 59 (97%) completed the randomized trial phase, 55 (90%) completed the 24-week follow-up, and 52 (85%) completed the one-year follow-up. Statistically significant between-group differences were found in favor of the combined experimental group for the fatigue-related quality of life, fitness outcomes, and overall physical activity. Paired comparisons confirmed significant within-group improvements for both groups from baseline to one-year follow-up across most outcomes. Discussion: A group-based combined physiotherapeutic and lower-body resistance exercise program was feasible and effective. Findings are limited to individuals who had undergone a surgical neck dissection procedure. Given the complexity of head and neck cancer, further pragmatic interdisciplinary research is warranted.

## 1. Introduction

In North America, head and neck cancers (HNCs) account for approximately 3% of all malignant tumors [1]. HNC results in considerable impairment of speech, swallowing, respiration, and cosmesis all radically altered by the cancer and/or cancer treatment [2,3]. Moreover, shoulder and neck dysfunction commonly occur following surgery [4,5], and individuals undergoing adjuvant cancer treatment are likely to experience significant weight loss as well as declines in muscular strength and physical functioning [6,7]. Thus, a strategic interdisciplinary rehabilitation approach is warranted to address the complexity of symptoms and maximize overall functioning and quality of life.

Individuals with HNC are encouraged to participate in exercise programs to aid in their recovery from cancer treatment [8]. However, there are well-known barriers to general exercise and physical activity among individuals with HNC including dry mouth, difficulty eating, weight loss, fear of injury, comorbidities, and treatment-related symptoms of pain and fatigue [9,10]. Indeed, in a recent systematic review of studies examining the benefit of general exercise interventions for individuals with HNC, the authors identified patient recruitment, retention, and adherence to programming as major concerns [10]. Based on the review findings, an interdisciplinary approach was recommended to address barriers specific to HNC, and tailoring of exercise programming to promote uptake and adherence [10].

Previous work conducted by our group demonstrated the benefit of targeted upper-extremity progressive resistance exercise training (PRET) for neck dissection-related pain and dysfunction. In our randomized controlled trial with an optional cross-over, fifty-two individuals with head and neck cancer who had undergone surgery were assigned randomly to PRET (*n* = 27) or a standardized physiotherapeutic protocol (*n* = 25) for 12 weeks [11]. The PRET program was found to significantly reduce shoulder pain and disability and improve upper extremity muscular strength and endurance. At a 12-month follow-up, participants who continued to follow the PRET program reported better neck dissection-related functioning and quality of life than those who did not continue with the program [12]. Although significant benefits were found from the PRET program for post-surgical shoulder outcomes, we found several barriers to clinical implementation—namely, the high cost of the one-on-one physical therapy supervision format, the need for specialized resistance exercise machines in the clinical setting, and the lack of benefit for fitness and functioning outcomes needed to facilitate a return to work. Moreover, our efforts to support adoption of exercise through community-based programming highlighted challenges with adherence and completion among HNC participants. Thus, given the high morbidity associated with HNC treatment, we aimed to evaluate a bridging program to support the transition from clinical rehabilitation services to community-based exercise programming [13].

The primary aims of this study were three-fold: (1) to assess the effectiveness of the addition of lower-body resistance exercise training to a physiotherapeutic shoulder and neck exercise protocol on symptoms of fatigue-related quality of life; (2) to evaluate the feasibility of delivering the intervention in a supervised group format; and (3) to examine the benefit of ongoing supported exercise over a one-year period.

## 2. Methods

### 2.1. Trial Design

**Figure 1:**
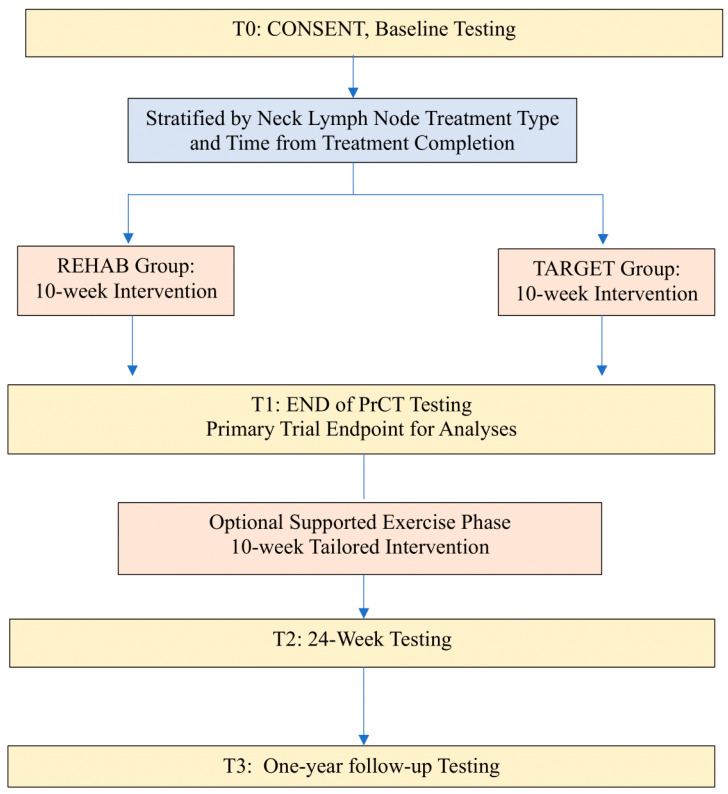
Study Schema.

The present study was a pragmatic randomized controlled trial (PrCT) examining outcomes from a combined physiotherapeutic and general physical exercise program. The design included the option for ongoing exercise support to progress participants to general exercise programming (Figure 1: Study Schema). The trial was registered at clinicaltrials.gov on 4 January 2016 (NCT02647021).

#### 2.1.1. Phase I: Pragmatic Randomized Controlled Trial Phase (Weeks 1–12)

This phase of the study involved a 10-week supervised group exercise program offered to all participants. Participants were required to attend sessions twice a week for 10 weeks. The purpose of this phase was to evaluate the feasibility and short-term effectiveness of the addition of lower-body resistance exercise to a physiotherapeutic exercise protocol focusing on the shoulder and neck. Participants were supervised by an interdisciplinary team that included a physical therapist, exercise specialist, and therapy assistant. The ratio of supervision (trainer:participant) for Phase I was 1:3.

#### 2.1.2. Phase II: Supported Exercise Transition Phase (Weeks 12–24)

Participants in both groups had the option to continue to attend group sessions and to transition to a general physical exercise program over a second 10-week period. For Phase II, the ratio of trainer:participant supervision was 1:5.

### 2.2. Eligibility Criteria

The inclusion criteria reflected the head and neck cancer populations seen in our clinical practice setting and included the following: (1) diagnosis of head and neck cancer (i.e., squamous cell carcinoma of the oral cavity, oropharynx, larynx, or hypopharynx), thyroid cancer, or melanoma; (2) neck cancer treatment included all variants of neck dissections including selective, modified, and radical procedures; or radiation therapy to lymph node regions in the neck; (3) Karnofsky Performance Status greater than or equal to 60% [14,15]; (4) no distant (M0) metastasis; (5) participants must have completed their head and neck/thyroid/melanoma cancer treatment (minimum 4 weeks post-treatment). Participants were excluded if they presented with medical illness or psychiatric illness, which, in the opinion of the investigators, would impact their ability to participate in exercise or interfere with follow-up.

### 2.3. Recruitment and Settings

Recruitment took place between April 2016 and March 2019. Potential participants were identified by their oncologist/surgeon at respective follow-up clinics at the University of Alberta Hospital and Cross Cancer Institute, or through the Oncology Rehabilitation Department of the Cross Cancer Institute. Individuals interested in taking part in this study were provided with a study pamphlet and were advised to contact the research team if interested in taking part. Prior to participation in this study, physician approval was obtained, and potential participants completed the Physical Activity Readiness Questionnaires (PAR-Q+) to determine the appropriateness of the exercise program. The study coordinator screened participants for eligibility. Interested participants were scheduled for a baseline visit to obtain written consent, determine final eligibility via exercise screening and testing, and complete the baseline assessment. The testing and intervention components of the trial were conducted in the Cancer Rehabilitation Clinic in Corbett Hall at the University of Alberta.

### 2.4. Randomization

Randomization was stratified by (1) the time from diagnosis (within 18 months) or late (greater than 18 months); and (2) the type of cervical lymph node treatment: radical neck dissection (i.e., spinal accessory nerve sacrificed), modified/selective neck dissection with spinal accessory nerve spared, or radiation therapy to the lymph nodes in the neck only (i.e., no surgery). In each stratum, participants were randomized in a one-on-one ratio to the REHAB group: a shoulder and neck protocol; or to the TARGET group: the shoulder and neck protocol with the addition of lower extremity resistance exercise training program.

### 2.5. Concealment of Allocation

An independent researcher generated the allocation sequence by using a computer-generated code. A block permutation procedure was used to generate the allocation sequence within each stratum. The allocation sequence and contents of the envelopes were enclosed in sequentially numbered and sealed (opaque) envelopes.

### 2.6. Protection from Sources of Bias

At each measurement point following the baseline assessment, including the end of the PrCT, 24-week and one-year follow-ups, an independent assessor performed the objective measurements. The independent assessor performing the end of the PrCT testing was blinded to group allocation. The independent assessor also administered the neck dissection impairment questionnaire and the FACT-Fatigue quality of life questionnaire. Exercise adherence was monitored by the research coordinator. Blinding of participants and the research coordinators was not possible. Study participants were free to withdraw from this study at any time but were invited to continue to attend for the 24-week and one-year follow-ups.

### 2.7. Outcomes—Collected at Baseline, Week 12, Week 24, and One Year

Baseline demographics and medical data were collected via participant interviews and abstraction from the electronic medical record at the Cross Cancer Institute.

### 2.8. Primary Outcome: Cancer-Related Fatigue

The primary outcome for this study was the change in the Functional Assessment of Cancer Therapy-Fatigue Scale (FACT-F), an outcome measure to assess the quality of life concerns related to fatigue from baseline to post-intervention [16,17].

### 2.9. Secondary Outcomes: Objective and Patient-Reported Measures

Objective physical outcome measures to inform optimal rehabilitation care included:

- Height, weight (calculation of body mass index).
- Aerobic endurance: a six-minute walk test (6MWT) was performed in a hallway using a 25 m distance [18].
- Flexibility: the sit-and-reach test was used to assess flexibility of the lower extremity [19,20].
- Shoulder range of motion (ROM) was measured following standardized procedures using a traditional goniometer [21,22].
- Muscle strength was assessed through measures of grip strength and by using the one-repetition maximum (1-RM) method for bench press, leg press, and seated row. The Jamar hydraulic hand dynamometer was used to measure grip strength and is considered a gold standard for measurement of grip strength [23].
- The 1-RM is recognized as the gold standard for assessing muscle strength and was implemented using the same exercise patterns and equipment that were used by participants during the exercise program [24]. The 1-RM is the highest weight that can be lifted once using proper form, a smooth motion, and without pain or other symptoms [24].
- Muscular endurance was assessed by using a submaximal seated row test. The weight for this test was set at 50% of the individual’s baseline 1-RM weight and the test was performed at a cadence of 22 repetitions per minute (set by a metronome) [11]. The maximum number of repetitions performed before falling behind the required cadence was recorded.

Patient-reported outcome measures with demonstrated validity and reliability included:

- Physical activity level was measured by the Godin Leisure Time Physical Activity Questionnaire [25,26,27].
- Neck dissection-related quality of life was measured by the Neck Dissection Impairment Index (NDII). The NDII is a valid and reliable instrument for assessing neck dissection impairment [28].
- Functional status was measured by the Trial Outcome Index of the Functional Assessment of Cancer Therapy–Fatigue Scale (sum of the Physical Well-Being, Functional Well-Being, and Fatigue Subscales) [29].
- Exercise adherence: attendance was taken at each exercise session.

### 2.10. Interventions

To facilitate clinical implementation, the exercise program was offered in a group setting with cohorts starting in January, April, and September of each year. This pragmatic format allowed for resource planning, transition to community-based programming, and flexibility for both the clinician and participant. Individuals in both groups received information on the importance of exercise following cancer treatment and how best to incorporate physical activity into their day-to-day lives. Participants were encouraged to progress their physical activity with the goal of achieving public health guidelines for physical activity (i.e., at least 150 min of moderate-intensity aerobic exercise each week) [30,31]. Details are shown in Table 1. TIDieR Checklist.

Group 1: REHAB—Therapeutic Protocol alone (active control arm)

The therapeutic protocol included:

(a) Neck and shoulder active and passive range of motion exercises;
(b) Shoulder-specific progressive resistance exercise training (PRET) program.

Group 2: TARGET—Therapeutic Protocol + Lower-Body Resistance Exercise

The combined protocol included:

(a) Neck and shoulder active and passive range of motion exercises;
(b) Shoulder-specific progressive resistance exercise training (PRET) program;
(c) Progressive resistance exercise training for the lower extremity.

### 2.11. Sample Size

Based on data from the 12-month follow-up of our previous trial, fifty-two HNC survivors were required to have an 80% chance of detecting, as significant at the 5% level, an increase in quality of life (FACT-F) of 3 points (standard deviation, 9) in the REHAB group and 10 points in the TARGET experimental group at the end of the PrCT. Based on an anticipated 15% drop-out/loss to follow-up, an additional eight participants were added for a total sample of 60 (i.e., 30 per group).

### 2.12. Statistical Analysis Plan

Descriptive statistics are presented for the medical and demographic characteristics and the study outcomes. Numbers and percentages were calculated and compared using the Chi-Square test between groups when appropriate to compare frequency distribution. Treatment effects for objective and patient-reported outcomes within each group were conducted using paired *t*-tests, and the analysis of covariance was used to investigate the between-group differences in the change score of the FACT-F and other objective outcome scores, adjusting for baseline value and time from treatment completion. We conducted a complete case analysis restricted to participants with complete data. All statistical tests were two-sided, and the significance level was set at *p* < 0.05.

### 2.13. Ethical Considerations

This study was approved by the Health Research Ethics Board: Cancer Committee (HREBA.CC-15.0167; approved 5 February 2016) and written consent was obtained from all subjects.

## 3. Results

### 3.1. Phase I: PrCT

**Figure 2:**
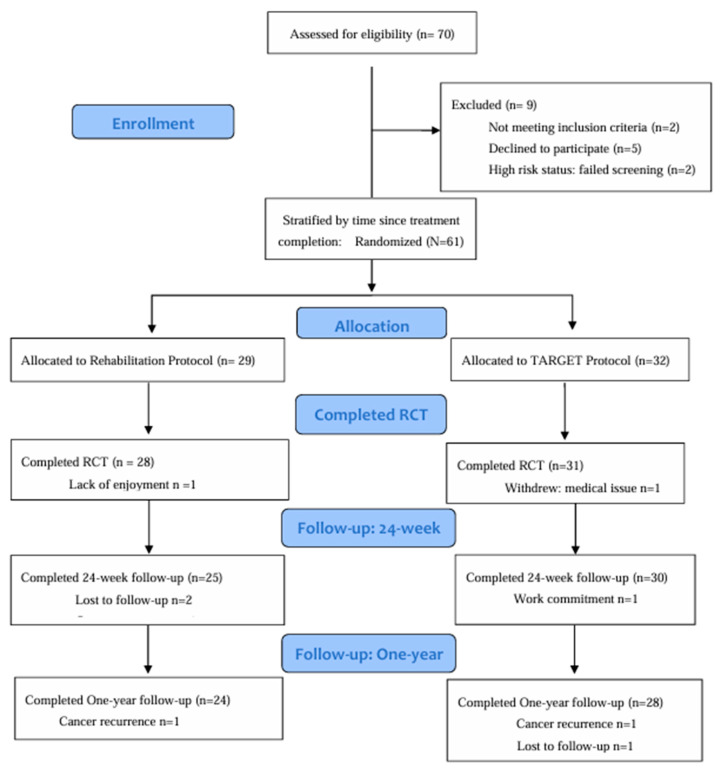
Study Flow.

This study took place between April 2016 and March 2020. A total of 70 individuals with HNC contacted the investigators interested in taking part in the trial (Figure 2: Study Flow). Of the 61 participants enrolled, 59 (97%) completed the PrCT (primary study endpoint), 55 (90%) completed the 24-week follow-up, and 52 (85%) completed the one-year follow-up. Participant characteristics are displayed in Table 2. For the PrCT, we present an intent-to-treat analysis based on the entire accrued population, and retained all outliers as they reflect variability inherent in the HNC population [32].

Among participants, the reported adherence was 92.2% (±15) for the REHAB group and 93.8% (±12) for the TARGET group (*p* = 0.665). Fifty-one (84%) participants opted to take part in the Phase II optional supported exercise, with 24 (83%) from the REHAB group and 27 (87%) from the TARGET group (*p* = 0.776). The primary reasons for not taking part in the supportive phase were related to travel distance to the center (*n* =2), not interested (*n* = 1), cancer recurrence (*n* =2), dental extractions (*n* = 1), and work-related commitments (*n* = 2). Adherence to the supportive phase ranged from 50% to 100%, with a mean of 95.4% in the REHAB group and 90.7% in the TARGET group (*p* = 0.239).

At the end of the PrCT phase, a statistically significant between-group difference was found in favor of the TARGET group for the primary outcome, the FACT-F scale (Table 3). TARGET was also superior to REHAB for the six-minute walk test distance, and all upper- and lower-body one-repetition maximum muscular strength tests. Both groups showed within-group improvements for the NDII, active shoulder abduction range of motion, one-repetition maximum seated row, and upper extremity endurance, whereas only the TARGET group showed significant within-group differences for the FACT-F scale, Trial Outcome Index, grip strength, and one-repetition maximum bench press. A significantly larger proportion of participants in the TARGET group versus the REHAB group were meeting physical activity guidelines at the end of the PrCT [48.4% versus 21.4%; *p* = 0.031]. No significant within- or between-group differences were found for changes in lower-body flexibility or body mass index, and no significant between-group differences were found for changes in grip strength.

### 3.2. Phase II: Supportive Exercise Transition

**Figure 3:**
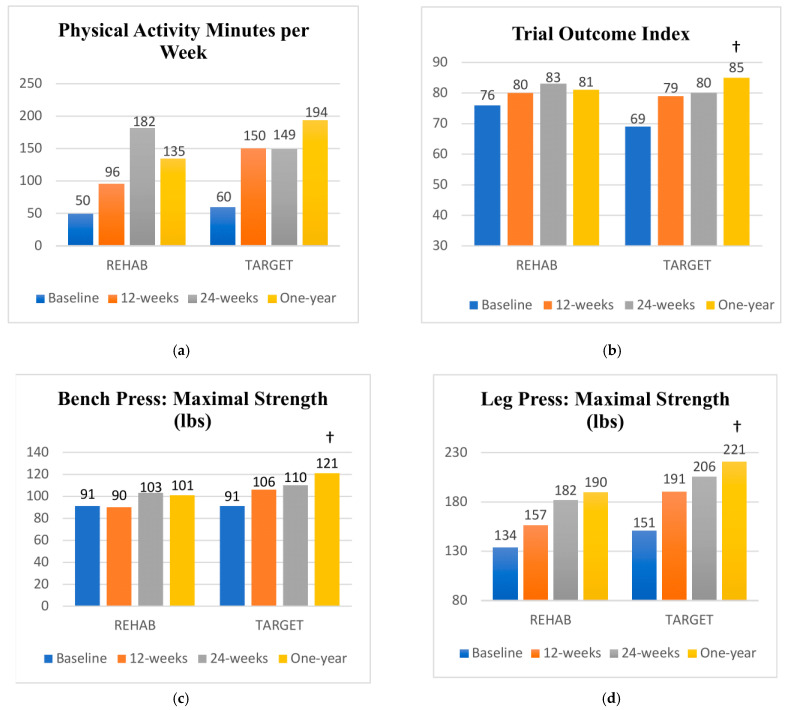
(**a**–**d**) Mean scores at each study time point; † significant between group difference in the change from baseline to one-year follow-up in favour of the TARGET group.

Participants in both groups continued to improve over the one-year follow-up (Supplementary Material: Tables S1 and S2). Paired comparisons confirmed significant within-group improvements in both intervention groups from baseline to one-year follow-up for the six-minute walk test, body mass index, NDII, active shoulder abduction range of motion, grip strength, sit-and-reach flexibility test, one-repetition maximum seated row, upper extremity endurance, and physical activity minutes (Figure 3a). The TARGET group showed within-group improvements for the FACT-F, Trial Outcome Index, as well as the one-repetition maximum bench press and leg press. There were no significant between-group differences in outcomes at the 24-week follow-up. Significant between-group differences were found in favor of the TARGET group for the change score from baseline to one-year for the Trial Outcome Index, and both the one-repetition maximum bench press and leg press (Figure 3b–d).

### 3.3. Adverse Events

No major or minor adverse events occurred related to study participation. Two minor musculoskeletal adverse events, both unrelated to study participation, were reported during the follow-up period (TARGET group: work-related back injury (*n* = 1); REHAB group: inguinal hernia related to heavy lifting at home (*n* = 1)). In each case, the injury resolved over time, and both participants completed the one-year follow-up testing.

## 4. Discussion

The main objective of the present PrCT was to assess the effectiveness of a combined physiotherapeutic and lower-body resistance exercise training protocol on fatigue-related quality of life as measured by the FACT-F. A secondary aim was to evaluate the feasibility of delivering the intervention in a supervised group format as well as to examine the benefit of ongoing supported exercise. Compared to the REHAB group, the TARGET group showed significantly greater improvement in the FACT-F and six-minute walk distance over the short-term, and significantly greater improvement in muscular strength scores and functional status over the long term. We found a medium to large effect size, suggesting that adding lower-body resistance exercise training to a physiotherapeutic regimen is both feasible and may support better long-term outcomes.

A recent meta-analysis examining adherence rates across chronic conditions reported that exercise studies involving individuals with cancer had greater variability in adherence and drop-outs than other disease groups [33]. Specific to HNC, previous reviews have highlighted the need for strategies to support the adoption and maintenance of an active lifestyle after cancer treatment [10,33]. Although intuitively, the prescription of additional exercise should result in larger fitness and functioning benefits, individuals with HNC may have poor exercise tolerance due to pain and fatigue, challenges with nutrition intake, and losses in lean body mass that occur with treatment, leading to a worsening of symptom burden [34]. The TARGET protocol included an additional three to five lower-body resistance exercises that were tailored to the individual’s strength level (taking ~10 more minutes per session), and participants were closely monitored for muscle soreness, increased fatigue, and weight loss, allowing the program to be modified as needed.

A strength of the current trial was the high reported adherence rate of participants of >92% in the PrCT phase and >90% in the supported exercise phase. Our adherence rates are similar to the rate of 93% reported in a systematic review of physical therapy interventions for HNC [35], and in the higher range of trials (45.2% to 93.1%) reported in a systematic review of exercise interventions for HNC [10]. Our interdisciplinary regimen allowed us to tailor the prescription to address disease and treatment-related symptomatology (i.e., shoulder pain and dysfunction) along with general physical functioning [33]. The high completion and adherence rates from our PrCT suggest the acceptability of the intervention among trial participants. The findings also support the positive short-term benefit seen from the addition of lower-body resistance exercise for increasing overall physical activity.

Little research has investigated the benefit of an interdisciplinary approach to rehabilitation in HNC. To our knowledge, this is the first trial to examine the effectiveness of a combined physiotherapeutic and lower-body resistance exercise intervention. Prior studies involving interdisciplinary approaches have focused on exercise coupled with nutrition [36]. Capozzi et al. (2012), in an exploratory RCT, examined the timing (either during or following treatment) and effect of a 12-week combined progressive resistance exercise training and nutrition intervention on body composition, fitness, and quality of life in 60 individuals with HNC. Although no significant between-group differences were found, a main effect of time was found for fitness outcomes and quality of life supporting improvement regardless of group assignment. Bye et al. (2020), in a systematic review and meta-analysis including 13 RCTs that examined nutrition and exercise interventions for HNC, reported significant improvements in physical function from trials of either exercise alone or combined exercise and nutrition interventions [10]. Improvements in body composition, however, were only found in studies examining nutrition interventions alone. As adequate nutrition is essential to restore muscular strength and address fatigue and quality of life, a multidimensional approach incorporating nutrition with physical therapy and exercise may further enhance outcomes [36].

We chose a pragmatic approach using a proven comparison intervention. As our current standard of care has substantial practice variation, the use of a comparison intervention allowed us to control for factors such as the natural course of recovery, attention, social interaction, and the participant–provider relationship [37]. The REHAB and TARGET interventions were delivered initially in small groups of three participants, reducing overall costs while allowing the staff to easily monitor the participants. The group format also provided the opportunity to bring individuals with HNC together to create their own supportive environment [38]. Not surprisingly, both groups showed improvements in shoulder and neck outcomes and experienced benefits over the one-year study period. The continued improvement seen in the treatment effect (relative to baseline) suggests promise from this strategy to bridge individuals with HNC from rehabilitation to general exercise and physical activity.

A primary limitation of this study was that once the REHAB group started Phase II, there was no longer a true comparison group, limiting our ability to evaluate the benefit of the combined intervention beyond the PrCT. As we were interested in investigating how to optimize recovery for individuals with HNC, we selected a pragmatic design with a broader inclusion of participants that reflected our clinical practice and chose relevant outcomes that could be easily captured in the clinical setting. Although the point estimates used for our sample size calculation aligned with our findings, the inclusion of a more heterogeneous participant population resulted in larger variability in outcomes than anticipated, limiting the statistical power of the analyses. Limits to external generalizability include a sample that was highly educated, largely of higher socioeconomic status, and primarily comprised individuals who had undergone a surgical neck dissection procedure. Despite the limitations, this PrCT provides insight into the feasibility and effectiveness of an interdisciplinary bridging program.

## 5. Conclusions

Our findings suggest that a combined physiotherapeutic and lower-body resistance exercise program delivered in a supervised group format was feasible and effective in addressing quality-of-life concerns related to fatigue, physical function, and muscular strength in individuals with HNC in the post-cancer treatment phase. Given the challenges and complexity of HNC, further pragmatic interdisciplinary research is warranted, and consideration should be given to integrating other supportive interventions such as nutrition.

## Figures and Tables

**Table 1:** TIDieR Checklist.

Intervention	REHAB Group	TARGET Group
**Why**	A physiotherapeutic and upper extremity PRET program will enhance the muscular strength of the scapular muscles and reduce patient-rated shoulder pain and disability	An intervention involving the addition of lower-body resistance exercises will enhance overall muscular strength and result in improved symptoms of cancer-related fatigue
**What: Materials**	Clinic: elastic bands, free weights, exercise machines (chest press, vertical bench, bicep curl, seated row, and triceps pushdown)	Clinic: elastic bands, free weights, exercise machines (chest press, vertical bench, bicep curl, seated row, and triceps pushdown; leg press/extension, hamstring curl) and mats (core exercises)
**What: Procedures**		
**Providers**	Interdisciplinary team: Physical therapist—oversight TA/QEP—intervention	Interdisciplinary team: Physical Therapist—oversight TA/QEP—intervention
**How**	Supervised group sessions: therapist-to-participant ratio of 1:3	Supervised group sessions: therapist-to-participant ratio of 1:3
**Where**	University-based Cancer Rehabilitation Clinic	University-based Cancer Rehabilitation Clinic
**Type**	Range of motion (ROM) for the neck and shoulders; upper extremity progressive resistance exercise training (PRET)	ROM for the neck and shoulders; upper extremity PRET; core, and lower extremity PRET
**Intensity**	2–4 (light to somewhat hard) on the 11-point Borg RPE Scale	[2–4 (light to somewhat hard) on the 11-point Borg RPE Scale]
**Progression**	Upper extremity: 30% progressed to 60% of 1-RM	Upper extremity: 30% progressed to 60% 1-RM; Lower extremity: 50% progressed to 80% of 1-RM
**Frequency**	Twice weekly	Twice Weekly
**Session time**	60 min per session	65–75 min per session
**Overall duration**	10 weeks	10 weeks
**Tailoring**	Adaptations to address spinal accessory nerve dysfunction, trapezius paresis/weakness, pain and fatigue, muscular stiffness, and to prevent adverse events	
**Trial fidelity**	Supervision by staff with training and experience in exercise oncology; Attendance tracked for number of completed exercise sessions; Monitoring of symptoms (e.g., fatigue, muscle soreness); Recording of adverse events.	

TA: therapy assistant; QEP: qualified exercise professional.

**Table 2:** Baseline demographic, medical, and behavioral profile of participants.

No. of Participants (%)			
Variable	Overall (N= 61)	Rehab Protocol (*n* = 29)	TARGET Protocol (*n* = 32)
Demographic profile			
Mean age (range), year	62.0 (28–86)	62.2 (28–86)	61.7 (39–84)
Female	22 (36%)	12 (41%)	10 (31%)
Married/Common Law	49 (80%)	20 (69%)	29 (90%)
Completed University	29 (48%)	17 (59%)	12 (38%)
Household Income > USD 80,000/year	30 (49%)	11 (38%)	19 (61%)
On disability	18 (30%)	9 (31%)	9 (28%)
Mean time from treatment (range), mo.	18.5 (1–148)	17.4 (3–138)	19.5 (1–148)
≤1 year	37 (61%)	17 (59%)	20 (63%)
>1 to 5 years	21 (34%)	11 (38%)	10 (31%)
>5 years	3 (5%)	1 (3%)	2 (6%)
Cancer Type			
Oral/oropharynx	42 (69%)	18 (62%)	24 (75%)
Larynx/nasopharynx	5 (8%)	4 (14%)	1 (3%)
Thyroid	6 (10%)	4 (14%)	2 (6%)
Other *	8 (13%)	3 (10%)	5 (16%)
Disease Stage			
I–III	37 (61%)	17 (59%)	20 (63%)
IV	24 (39%)	12 (41%)	12 (38%)
Neck Dissection (Total)	55 (90%)	27 (93%)	28 (88%)
Bilateral Neck Dissection	44 (72%)	21 (72%)	23 (72%)
Neck Dissection Classification (side with most extensive dissection)			
RND	6 (10%)	3 (10%)	3 (9%)
MRND	32 (52%)	13 (45%)	19 (59%)
SND (Level 5 spared)	17 (28%)	11 (38%)	6 (19%)
Radiation to lymph node regions	6 (10%)	2 (7%)	4 (12%)
Radiation Therapy (Total)	54 (89%)	27 (93%)	27 (84%)
Adjuvant Radiation Therapy	31 (51%)	17 (59%)	14 (44%)
Concurrent with Chemotherapy	23 (38%)	10 (34%)	13 (41%)
Chemotherapy Type (Total)			
Cisplatin	17 (28%)	7 (24%)	10 (31%)
Cisplatin and Carboplatin	2 (3%)	1 (3%)	1 (3%)
Carboplatin	3 (5%)	1 (3%)	2 (6%)
Immunotherapy **	2 (3%)	2 (7%)	0 (0%)
Other Medical			
Mean BMI (range)	24.6 (17–45.5)	24.1 (17–32)	25.1 (17–45.5)
HPV Positive	29 (69%)	10 (56%)	19 (59%)
Behavioral Profile			
Current exerciser ***	9 (15%)	3 (10%)	6 (19%)
Never smoker	24 (39%)	10 (34%)	14 (44%)
Non-drinker	20 (33%)	11 (38%)	9 (28%)

* Unknown primary (*n* = 6), melanoma (*n* = 1), salivary gland (*n* = 1); ** immunotherapy for melanoma (*n* = 1), SCC larynx (*n* = 1); *** meeting or exceeding public health recommendations of 150 min of moderate to vigorous activity per week.

**Table 3:** Self-reported quality of life and objective outcomes.

	T0: Baseline	T1: End of PrCT	Unadjusted Within-Group Mean Difference: T0 to T1	Unadjusted Between-Group Mean Difference: T0 to T1	Adjusted ^1^ Between-Group Mean Difference: T0 to T1
Outcome	Mean (SD)	Mean (SD)	Mean Change [95% CI]	Effect size [95% CI]	Mean Change [95% CI]
FACT-F (0–160)					
REHAB Group	116.4 (21.4)	119.4 (20.6)	1.5 [−2.9, 5.9]		
TARGET Group	107.3 (25.2)	117.5 (20.3)	9.7 [5.5, 13.9] *	0.7 [0.17, 1.22]	+5.7 [0, 11.7] †
Trial Outcome Index (0–108)					
REHAB Group	76.3 (19.0)	79.5 (18.5)	1.96 [−3.2, 7.1]		+4.75 [−1.5, 10.9]
TARGET Group	68.6 (20.9)	78.5 (14.6)	10.5 [5.6, 15.3] *	0.6 [0.1, 1.1]	
Neck Dissection Impairment Index (0–100)					
REHAB Group	55.6 (23.0)	69.3 (20.1)	13.7 [7.0, 20.3] *		+2.5 [−5.6, 10.6]
TARGET Group	52.6 (21.2)	65.5 (20.3)	11.7 [5.4, 18.0] *	+0.1 [−0.4, 0.6]	
Six-Minute Walk Test (m)					
REHAB Group	485.4 (96.1)	512.8 (92.4)	27.8 [6.5, 49.1] *		
TARGET Group	484.2 (100.4)	543.0 (91.5)	57.3 [37.0, 77.5] *	0.5 [0.0, 1.0]	+29.8 [2.7, 56.9] †
1-RM Leg Press (lbs) ^2^					
REHAB Group	134.2 (48.9)	156.6 (57.2)	20.1 [5.5, 34.6] *		
TARGET Group	150.9 (53.1)	190.8 (80.4)	43.1 [29.6, 56.6] *	0.6 [0.1, 1.2]	+21.1 [0.7, 41.6] †
Lower-Body Flexibility (cm)					
REHAB Group	13.7 (12.0)	16.0 (11.9)	2.1 [−3.4, 4.7]		+1.2 [−2.7, 5.1]
TARGET Group	10.6 (11.6)	12.1 (12.4)	1.5 [−4.7, 3.9]	0.1 [−0.4, 0.6]	
Body Mass Index					
REHAB Group	24.3 (4.1)	24.7 (4.3)	0.2 [−0.2, 0.7]		
TARGET Group	25.1 (5.4)	25.7 (6.3)	0.3 [−0.1, 0.8]	0.1 [−0.4, 0.6]	+0.08 [−0.56, 0.71]
Active Shoulder Abduction (Degrees)					
REHAB Group	108.7 (38.9)	135.6 (32.4)	28.4 [17.5, 39.3] *		−7.6 [−20.7, 5.6]
TARGET Group	107.7 (33.9)	128.8 (33.4)	20.2 [9.8, 30.5] *	−0.3 [−0.8, 0.2]	
1-RM Bench Press (lbs)					
REHAB Group	91.2 (46.6)	90.1 (35.7)	−2.7 [−11.6, 6.3]		
TARGET Group	90.5 (48.4)	105.6 (47.3)	13.1 [4.7, 21.6] *	+0.7 [0.1, 1.2]	+15.8 [4.8, 26.9] †
1-RM Seated Row (lbs)					
REHAB Group	93.7 (46.6)	123.9 (54.9)	27.4 [17.0, 37.8] *		
TARGET Group	98.0 (57.7)	144.1 (63.8)	43.4 [33.5, 53.3] *	+0.6 [0.1, 1.1]	+16.2 [2.1, 30.3] †
UE Endurance (reps @ 50% 1-RM)					
REHAB Group	20.1 (5.6)	29.9 (10.1)	10.0 [5.9, 14.1] *		
TARGET Group	21.0 (9.3)	31.6 (14.0)	10.6 [6.7, 14.4] *	+0.1 [−0.5, 0.6]	+1.0 [−4.6, 6.6]
Grip Strength (lbs)					
REHAB Group	65.7 (21.6)	69.7 (21.6)	3.7 [−1.9, 9.3]		
TARGET Group	73.6 (24.3)	75.9 (27.8)	5.4 [2.9, 8.3] *	+2.1 [−1.8, 6.0]	−0.6 [−7.1, 8.3]

^1^ Adjusting for time from treatment and baseline score; SD: standard deviation; CI: confidence interval; 1-RM: one-repetition maximum; UE: upper extremity * significant within-group change *p* < 0.05; † significant between-group change *p* < 0.05; Leg Press ^2^: Rehab *n* = 25; TARGET *n* = 29.

## Data Availability

Data will be accessible on request via the University of Alberta Cancer Rehabilitation Clinic Dataverse at: https://borealisdata.ca/privateurl.xhtml?token=077a9a24-fbed-409a-b69f-0891158bf684.; Accessed 20 April 2024.

## Supplementary Materials

The following supporting information can be downloaded at: https://www.mdpi.com/article/10.3390/cancers16091758/s1, Table S1: Quality of Life: Fatigue, Physical Functioning, and Body Composition Outcomes T0 to T4; Table S2: Neck Dissection and Upper Extremity Outcomes T0 to T4.
