# Supplementary material for: Building a Bridge to Community: A Pragmatic Randomized Trial Examining a Combined Physical Therapy and Resistance Exercise Intervention for People after Head and Neck Cancer
Source: Cancers (Basel). 2024 May 1;16(9):1758. doi: 10.3390/cancers16091758 (PMC11083025; doi:10.3390/cancers16091758)
Supplement: Supplementary file 1 [file cancers-16-01758-s001.zip › cancers-2969663-supplementary.pdf]

Table S1. Quality of Life: Fatigue, Physical Functioning, and Body Composition Outcomes T0 to T4

	**T0: Baseline**	**T1: End of PrCT**	**T3: 24-week Follow-up**	**T4: One-year Follow-up**	**Adjusted between-group mean difference: T0 to T4**
**Outcome**	**Mean (SD)**	**Mean (SD)**	**Mean (SD)**	**Mean (SD)**	**REHAB vs TARGET Mean Change [95% CI]**
FACT-F (0-160)					
REHAB Group	116.4 (21.4)	119.4 (20.6)	122.7 (19.3)	122.1 (24.4)	
TARGET Group	107.3 (25.2)	117.5 (20.3)	120.1 (21.3)	123.2 (21.0)*	+7.5 [-3.8, 18.8]
Trial Outcome Index					
REHAB Group	76.3 (19.0)	79.5 (18.5)	83.0 (16.8)	80.7 (22.4)	
TARGET Group	68.6 (20.9)	78.5 (14.6)	79.8 (19.1)	84.7 (14.8)*	+8.2 [0.7, 15.8]†
Six-Minute Walk Test (m)					
REHAB Group	485.4 (96.1)	512.8 (92.4)	537.9 (96.4)	542.9 (112.4)*	
TARGET Group	484.2 (100.4)	543.0 (91.5)	551.3 (108.7)	561.1 (132.2)*	+15.1 [-31.3, 61.6]
1-RM Leg Press (lbs)					
REHAB Group	134.2 (48.9)	156.6 (57.2)	182.0 (67.4)	189.6 (79.2)	
TARGET Group	150.9 (53.1)	190.8 (80.4)	204.9 (68.1)	222.4 (80.0)*	+24.9 [41, 45.7]†
Lower Body Flexibility (cm)					
REHAB Group	13.7 (12.0)	16.0 (11.9)	17.4 (11.1)	18.3 (11.6)*	
TARGET Group	10.6 (11.6)	12.1 (12.4)	14.2 (11.9)	13.7 (12.8)*	-0.6 [-4.7, 3.6]
Body Mass Index					
REHAB Group	24.3 (4.1)	24.7 (4.4)	24.8 (4.2)	25.0 (4.1)*	
TARGET Group	25.1 (5.4)	25.7 (6.3)	25.9 (6.7)	26.2 (6.3)*	-0.08 [-1.0, 0.9]

[1]*Adjusting for time from treatment and baseline score; SD: standard deviation; CI: confidence interval; 1-RM: one-repetition maximum; * significant within group change p < 0.05; † significant between group change p < 0.05*

Table S2. Neck Dissection and Upper Extremity Outcomes T0 to T4

	T0: Baseline	T1: 12-week End of RCT	T3: 24-week Follow-up	T4: One-year Follow-up	Adjusted between-group mean difference: T0 to T4
**Outcome**	**Mean (SD)**	**Mean (SD)**	**Mean (SD)**	**Mean (SD)**	**REHAB vs TARGET Mean Change [95% CI]**
NDII (0-100)					
REHAB Group	55.6 (23.0)	69.3 (20.1)	68.8 (20.4)	72.1 (21.0)*	
TARGET Group	52.6 (21.2)	65.5 (20.3)	68.4 (19.4)	70.3 (23.6)*	+2.5 [-7.0, 12.1]
Active Shoulder Abduction (Degrees)					
REHAB Group	108.7 (38.9)	135.6 (32.4)	138.5 (31.6)	139.6 (27.9)*	
TARGET Group	107.7 (33.9)	128.8 (33.4)	131.4 (33.3)	134.5 (31.0)*	-1.0 [-15.3, 13.3]
1 RM Bench Press (lbs)					
REHAB Group	91.2 (46.6)	90.1 (35.7)	103.3 (38.5)	100.8 (43.2)	
TARGET Group	90.5 (48.4)	105.6 (47.3)	110.1 (46.3)	120.5 (47.9)*	+23.6 [6.6, 40.6]†
1 RM Seated Row (lbs)					
REHAB Group	93.7 (46.6)	123.9 (54.9)	134.1 (60.7)	144.1 (62.4)*	
TARGET Group	98.0 (57.7)	144.1 (63.8)	153.2 (66.3)	161.8 (76.0)*	+11.4 [-14.0, 36.8]
UE Endurance (reps @ 50% 1RM)					
REHAB Group	20.1 (5.6)	29.9 (10.1)	32.0 (15.2)	32.1 (14.3)*	
TARGET Group	21.0 (9.3)	31.6 (14.0)	36.0 (16.0)	33.2 (14.9)*	-0.08 [-8.5, 8.4]
Grip Strength (kgs)					
REHAB Group	65.7 (21.6)	69.7 (21.6)	71.1 (22.0)	72.7 (24.8)*	
TARGET Group	73.6 (24.3)	78.3 (27.8)	81.5 (27.3)	83.0 (26.5)*	+1.22 [-4.0, 6.5]
Physical Activity Minutes/ Week					
REHAB Group	49.9 (141.5)	96.4 (124.1)	184.8 (172.7)	127.6 (122.7)*	
TARGET Group	67.9 (103.6)	149.7 (130.0)	147.3 (132.1)	197.3 (203.1)*	+34.9 [-58.1, 127.9]

[1]*Adjusting for time from treatment and baseline score; SD: standard deviation; CI: confidence interval; 1-RM: one-repetition maximum; * significant within group change p < 0.05; † significant between group change p < 0.05*
